# Getting research into policy - Herpes simplex virus type-2 (HSV-2) treatment and HIV infection: international guidelines formulation and the case of Ghana

**DOI:** 10.1186/1478-4505-9-S1-S5

**Published:** 2011-06-16

**Authors:** H Burris, J Parkhurst, Y Adu-Sarkodie, P Mayaud

**Affiliations:** 1 London School of Hygiene & Tropical Medicine, London, UK; 2 School of Medical Sciences, Kwame Nkrumah University of Sciences & Technology, Kumasi, Ghana; 3 Currently employed by FHI - formerly Family Health International

## Abstract

**Background:**

Observational epidemiological and biological data indicate clear synergies between Herpes simplex virus type 2 (HSV-2) and HIV, whereby HSV-2 enhances the potential for HIV acquisition or transmission. In 2001, the World Health Organization (WHO) launched a call for research into the possibilities of disrupting this cofactor effect through the use of antiherpetic therapy. A WHO Expert Meeting was convened in 2008 to review the research results. The results of the trials were mostly inconclusive or showed no impact. However, the WHO syndromic management treatment guidelines were modified to include acyclovir as first line therapy to treat genital ulcer disease on the basis of the high prevalence of HSV-2 in most settings, impact and cost-benefit of treatment on ulcer healing and quality of life among patients.

**Methods:**

This paper examines the process through which the evidence related to HIV–HSV-2 interactions influenced policy at the international level and then the mechanism of international to national policy transfer, with Ghana as a case study. To better understand the context within which national policy change occurs, special attention was paid to the relationships between researchers and policy-makers as integral to the process of getting evidence into policy. Data from this study were then collected through interviews conducted with researchers, program managers and policy-makers working in sexual health/STI at the 2008 WHO Expert Meeting in Montreux, Switzerland, and in Accra, Ghana.

**Results:**

The major findings of this study indicate that investigations into HSV-2 as a cofactor of HIV generated the political will necessary to reform HSV-2 treatment policy. Playing a pivotal role at both the international level and within the Ghanaian policy context were ‘policy networks’ formed either formally (WHO) or informally (Ghana) around an issue area. These networks of professionals serve as the primary conduit of information between researchers and policy-makers. Donor influence was cited as the single strongest impetus and impediment to policy change nationally.

**Conclusions:**

Policy networks may serve as the primary driving force of change in both international context and in the case of Ghana. Communication among researchers and policy-makers is critical for uptake of evidence and opportunities may exist to formalize policy networks and engage donors in a productive and ethical way.

## Background

The process by which evidence generated from research is incorporated into policy (at both national and international levels) and practice is not always well understood, particularly in low income countries. While the dominant discourse in public health calls for evidence based policy —where evidence stands above local interests [1,2] — numerous policy analysis based models emphasize the importance of local context in shaping policy change or the uptake of evidence itself [3,4]. The overall purpose of this study was to evaluate the process of incorporating evidence from international or national research into both international and national policies/guidelines, in order to tease out the critical elements that determine likelihood of research uptake in these two international and national contexts respectively. This study focuses on case studies of incorporating management of herpes simplex virus type-2 (HSV-2) into genital ulcer disease (GUD) treatment guidelines at the World Health Organization (WHO, international level) and in Ghana (national level).

### HSV-2 and HIV synergies

Sexual acquisition of infection with HSV-2 (and sometimes HSV type-1) causes genital herpes, a chronic, lifelong disease, which manifests itself by recurrent ulcer episodes and frequent viral shedding on genital mucosal surfaces. HSV-2 is one of the most common sexually transmitted infections (STI) worldwide [5-7]. A number of clinical, epidemiological and biological studies have shown that HSV-2 is strongly associated with increased rates of HIV acquisition [8,9], and viral shedding in HSV-2/HIV co-infected individuals, thereby increasing their infectious potential for transmission [10-13]. Moreover, HIV infection alters the natural history of HSV-2 infection and severely immune-suppressed co-infected patients may experience more frequent, severe or prolonged symptomatic recurrences [14,15] as well as increased frequency of HSV-2 genital shedding [11,16-18], facilitating the sexual transmission of either virus. These reciprocal and synergistic relationships underscore the importance of controlling HSV-2 for HIV prevention [15,19].

### HSV-2 management: international guidelines

Acyclovir is an anti-herpetic drug that shortens the duration of HSV-2 clinical episodes if taken early, and prevents recurrences if taken over longer periods of time. However, this antiviral cannot cure genital herpes. It has been used widely in high income countries and has a good safety and efficacy profile [20]. WHO recommends that low resource settings manage GUD and other STI syndromes using the syndromic approach, which provides presumptive treatment for all possible treatable causes of the syndrome without the need to carry out expensive or time-consuming diagnostic tests. For a long time, the recommendation only included treatment of curable bacterial infections, not viral infections, such as HSV-2. However, since HSV-2 has become the most common GUD aetiology, and ulcers caused by HSV-2 are not necessarily distinguishable from other types of genital ulcers, particularly in HIV positive individuals, WHO recommended in 2003 to include acyclovir in the cocktail of drugs prescribed to GUD patients, with the provision that it should be supplied in settings where at least 30% of GUD are caused by HSV-2 [21]. Few nations have this country-level data. Moreover, there has been great reluctance in resource-limited countries to treat HSV-2 as it is perceived to be a self-limiting disease (at least in immuno-competent individuals), treatment is not curative as episodes are typically recurrent, there is a lack of awareness among providers and a lack of access to anti-herpetic drugs at the lower echelons of health care systems.

Prior to the addition of acyclovir to the management guidelines, WHO had convened a meeting of international researchers in 2001 to examine the role of herpes on the HIV epidemic in developing countries [22]. The meeting resulted in a call for research into the possibility of disrupting the observed co-factor effect of HSV-2 with the use of anti-herpetic therapy. Randomized controlled trials were set up in Asia, sub-Saharan Africa, Latin America and the United States to determine the impact of either short-term episodic treatment of genital ulcers or suppressive HSV therapy taken daily over several months, and included HIV-infected and uninfected populations. While the trials were in progress, the Global Strategy for the Prevention and Control of STIs (2006-2015) [23] was adopted at the World Health Assembly in Geneva in June 2006. The Strategy called, inter alia, for greater control of HSV-2 infection. By April 2008, all but one trial had come to an end. The results of the trials, as well as other biological, modeling and economic analyses, were presented at a WHO Expert Meeting in Montreux, Switzerland, which informed the revision of the International STI Treatment Guidelines.

The research found that 1) HSV-2 episodic therapy was marginally beneficial in some, but not all patient groups and settings, in terms of ulcer healing and reduction of HIV levels in the genital tract [24-26]; 2) HSV-2 suppressive therapy did not appear to prevent HIV acquisition in two large trials [27,28]; 3) HSV-2 suppressive therapy generally decreased HIV levels in plasma and genital secretions in most studies [10,13,29-33]; but 4) this was not enough to decrease HIV transmission between partners in HIV serodiscordant couples [34] (this final landmark study ended one year after the Montreux meeting and was published two years later). Despite these globally disappointing results, a recommendation was made during the Expert Meeting to modify the WHO GUD syndromic management guidelines to include anti-herpetic therapy in the syndromic management cocktail, without a prevalence threshold. The decision was based on high HSV-2 prevalence among GUD patients in all settings, clinical benefits to those treated, and a potentially favorable cost-benefit profile. Addition of acyclovir was found to increase the number of ulcers correctly treated thereby reducing the cost per ulcer treated, even when not taking into account the potential for for loss of productivity due to herpetic outbreaks and the increased rate of HIV acquisition among HSV-2 positive individuals [35].

### Ghanaian national policy

At the time of this study in July 2008, acyclovir was available in Ghana but only prescribed in the private sector. Meanwhile, Ghana was still employing the pre-2003 WHO GUD syndromic management guidelines which did not include acyclovir [36]. Select physicians, those with more resources, occasionally gave acyclovir as part of their treatment for GUD, but this was a rare occurrence. Ghanaian health officials cited competing health priorities and the chronic nature of herpes as obstacles to guideline changes, despite the quality of life argument to herpes suppression and the potential benefit to Ghanaian workforce productivity.

## Methods

The objectives of this study were: (1) to evaluate the process by which findings of trials into the synergy between HSV-2 and HIV on GUD influenced guidelines formulated by the WHO, and (2) to investigate the policy transfer process from international guideline formulation to policy development in Ghana. Both of these policy change mechanisms have been investigated based on theoretical frameworks of the policy process and the ways evidence may be taken up into policy. Also under examination was the relationship between research conducted locally (within Ghana) and national policy development, paying special attention to the Ghanaian policy context in an effort to better understand the climate within which policy change occurs at a national level.

A first set of in-depth interviews (n=11) was conducted informally with attendees of the WHO Expert Meeting to update the GUD syndromic management guidelines in Montreux, Switzerland, in April 2008. Interviewees included senior WHO officials, WHO program staff and researchers from across the globe. Oral consent was obtained based on conditions of anonymity and all interviews were conducted by a single interviewer (HB). A second set of interviews (n=8) was conducted formally with high-ranking government officials, leaders in the non-profit public health sector and distinguished researchers all working in the field of sexual and reproductive health, in Accra, Ghana, during July 2008. Interviews were conducted by the same interviewer (HB) in the presence of a senior Ghanaian STI researcher (YAS). Written consent was obtained from all interviewees. Interview questions were scripted and strictly adhered to for consistency. Interviews were conducted in English, tape-recorded and transcribed. The data from both sets of interviews were analyzed based on trend analysis. With a fairly small number of interviewees at the two sites, it was possible to complete a manual trend analysis by comparing the transcribed interviewee responses in order to identify the prevailing themes.

### Selection of case study

This research was commissioned by the DFID-funded Research Project Consortium (RPC) on Research and Capacity Building in Sexual & Reproductive Health and HIV in Developing Countries. Members of the RPC were personally involved in many of the HSV-HIV trials, including one conducted in Ghana [18,24] and they presented their findings at the Montreux meeting. The Ghana trial results had also been presented earlier (June 2006) to national stakeholders in Ghana, but no change in management guidelines was introduced then, although it was found that HSV-2 accounted for over 50% of genital ulcers in Ghana, thereby highlighting its public health importance [18]. The link to HIV also influenced selection of the case study, as international attention paid to HIV prevention and care in recent years has been noted for creating a ‘policy window’ for change in relation to the treatment of potential cofactor infections, particularly in parts of Africa which are greatly affected by the HIV epidemic [37].

### Theoretical frameworks for the evidence-policy interface

There are many theories about the way in which evidence generated from research is (or is not) incorporated into policy. The presence or expectation of an evidence base to a policy or practice has become commonplace, specifically in the public health field which tends to value evidence above ideological concerns [1,2]. However, insights into evidence and policy from a policy analysis perspective recognize the importance of other elements beyond the evidence itself as influential in shaping how research findings get taken up in decision making [38]. These models point to the importance of multiple competing political factors shaping policy-making [39], and often emphasize the links between key networks connecting researchers and policy makers [3]. In this case study, exploration has been dissected into three interconnected relationships to provide a framework to guide research and analysis: (1) evidence to policy theories, (2) actors and policy networks, and (3) international to national policy transfer theories.

#### Evidence to policy theories

A number of frameworks have been proposed to explain the research-policy interface. One of the most straightforward is the linear/rationalist model, which views policy change as pragmatic problem-solving with evidence providing the basis for policy decisions [40]. In this conceptualization, reviewing relevant evidence is all that is needed to guide policy to a correct decision. When evidence is not used as it should be, the problem is seen as a technical one – a breakdown in communication between the ‘two-worlds’ of researchers and policy makers. The ‘two-worlds’ view [41] particularly focuses on the different needs and expectations of researchers and policy-makers. The world of researchers is depicted as scientifically complex and places an emphasis on process and purity of findings. The world of policy-makers, on the other hand, wants certainty, timeliness and simplicity. Lomas describes the tension as “decision-makers accuse researchers of irrelevant, poorly communicated ‘products’; researchers accuse decision-makers of political expediency that results in irrational outcomes” [42].

This rationalist view, however, strikingly ignores politics and political interest in the research to policy continuum. Political theorists emphasize the political space, and typically point to the multiple competing elements which can influence policy makers. Lin, for instance, refers to three competing ‘rationalities’ in decision making – ‘technical’ rationalities (such as those driven by evidence) must compete with ‘political’ and ‘cultural’ rationalities (demands) [38]. Other authors similarly emphasize that policy making is not a simple point of decision making, but one which occurs in a broader context (with broader issues influencing decision making).

From these perspectives, the nature of the policy making body and its competing demands may be highly relevant to shaping the uptake of research into policy. International organizations, such as the WHO, are expected to have fewer competing political influences and a more homogenous cultural view of the importance of evidence in shaping health policy. Finally, the context of policy making would be insulated from many other issues due to the WHO’s narrow focus on healthcare. National governments, however, may show great variety in all these issues. The importance of research evidence in competition with other demands on policy makers, and the changing contexts and importance of health decisions may be very locally specific, and play a great role in shaping the uptake of evidence for any given case.

#### Actors and policy networks

Many health policy and research-to-policy theories, place actors and networks of actors centrally in explaining policy change [3,4], highlighting the importance of networks in influencing public policy outcomes [43]. Networks can take various forms in these works, including ‘epistemic communities’ of scientific or disciplinary experts who have access to policy-makers, or cross-cutting ‘policy networks’ linking actors representing government, economic and professional interests involved in a specific decision making structure [43,44]. Haas in particular contends that it is the informal networks that exist among and around actors that are key to bridging the research-policy gap at all levels [45]. Information flows through these communities freely, and if the community includes policy-makers or other influential persons (as well as researchers, or other custodians of evidence), the community itself can serve to exert pressure on those policy-makers, thus skirting tiresome bureaucratic channels [45]. Haas notes that policy communities in general may be difficult to trace, so their influence in policy development may be underestimated. In international organizations such as the WHO, however, these networks are much more evident and are often institutionally structured into decision making processes. One would expect to see clear and regular contact between researchers and decision makers in such an institution. At national levels, however, the relationships may be more variable – changing over time and according to the nature of the issue. As such, investigation is needed at the national level to understand how and when such networks play a role in policy change.

The RAPID (Research and Policy in Developing Countries) programme of the Overseas Development Institute reviewed 50 case studies to examine these processes. Their conceptual framework contends that there is an integral interconnectivity between the political context (political structures/processes, institutional pressures, prevailing concepts, policy streams and windows), the evidence (credibility, methods, relevance, use, how the message is packaged and communicated), and links (between policy-makers and other stakeholders, relationships, voice trust, networks, the media and other intermediaries) [46]. This all-inclusive analysis then places these three factors, or overarching themes, in the context of external influences, including economic and cultural influences among others [46]. The degree to which each of the factors takes center stage in terms of driving, impeding or simply engaging in research-to-policy is fluid throughout the process and highly case-specific; however, networks of actors remain central to the landscape of change in every instance.

#### International to national policy transfer theories

The complexities of policy making at a national level can further be illustrated by the processes in which international recommendations are transferred to the local level. Rather than a simple process of copying higher level guidelines, often a more iterative process exists where pieces of information are taken up, interpreted and adapted [47]. This process can be illustrated through specific country case studies. Based on a study of policy transfer of tuberculosis and STI management in Mozambique [48] for instance, it was found that policies do not appear to follow one transfer mechanism, but rather, policy formulation requires many “loops” that rely on both bottom-up and top-down dissemination. According to another global perspective case study of STI management [49], international-to-national policy transfer results from a process described by Kingdon’s famous ‘’streams” model which may be used to analyze policy change at local, national and international levels [50]. The model focuses on agenda setting and timing as the key components to policy reform. The so-called streams consist of (1) a problem stream – linked to identification or recognition of problems, (2) a policy stream – encompassing feasible policy solutions, and (3) a politics stream – reflecting the political will to address an issue [50]. A policy window is seen to occur when these three streams ‘intersect’ in time. That is to say when a problem is recognized, a solution is available, and political will is strong enough. It is then that policy reform is introduced. This window presents itself as the focal point for researchers hoping to influence policy, or the point at which evidence can be incorporated into policy. This window of opportunity may be the result of an organic process, but it may also be induced by well-placed actors (known as ‘policy entrepreneurs’) who recognize when time might be ripe for policy change [50]. Network theorists also see networks as playing key roles in international to national policy transfer [44], and it can be conceptualized that these policy entrepreneurs typically need to be members of important policy networks.

As a whole, then, there are numerous theories and models of the process of policy change, and of the uptake of research into policy. Those often used in public health discourse tend to see getting research into policy as a technical exercise— bridging the gap between evidence and policy circles. Those deriving from policy analysis perspectives, however, typically emphasize the complexity of factors influencing decisions and decision-makers. While diverse, the core of many of these theories have to do with political issues, competing interests, and policy processes that only enable change when particular conditions are met. In both sets of views, key actors and networks play important roles connecting researchers to policy-makers, pushing technical considerations in debates, and identifying windows of opportunity for policy change. Our study uses these insights to compare the evidence to policy process in the sexual health/STI field in both international (WHO) and national (Ghana) contexts.

## Results

### Influencing international policy: the WHO GUD syndromic management guidelines

Common among all Montreux interviewees is the belief that a ‘policy window’ was a key determinant for the inclusion of acyclovir in the GUD syndromic management guidelines. As suggested earlier, it would not be illogical to assume that the power of observational data alone may be more influential in the context of the WHO than in a national setting. It was not, however, found to be sufficient. One policy-maker in Montreux noted that “evidence is not sufficient: facts are facts, perception is reality” and went on to say that “even if there is evidence for a change in policy, it will not matter until it is perceived that it should matter.” This indicates that even in a context such as the WHO which focuses on best practices in health, there are multiple factors affecting the uptake of evidence. In line with Kingdon’s Three Streams Model, the observance of a synergy between HIV and HSV-2 (problem stream) combined with the international attention and donor focus on HIV (politics stream) served to push HSV-2 on to the international agenda. As stated by one WHO policy-maker, “without these trials [into disrupting the co-factor effect between herpes and HIV] we would not even be discussing herpes or thinking about making acyclovir standard practice.” It was highly anticipated that the trial results would provide evidence for an effective intervention (policy stream), thereby satisfying Kingdon’s prerequisites for the creation of a policy window. Interviewees agreed that the momentum generated through the potential to impact HIV transmission and acquisition through addressing HSV-2 allowed for inclusion of acyclovir in the guidelines despite the trials’ findings showing no impact on HIV transmission: it provided a platform to those who sought to “treat herpes for herpes sake,” commented one researcher.

As noted above, the initial impetus for policy change with regards to HSV-2 was a bottom-up process in which observational data in a few countries triggered international investigation. Once evidence of a synergy between the two viruses had accumulated through in-country observational data and some international reviews and meta-analyses, the ‘policy network’ of researchers, program managers and policy-makers who were to later comprise the 2008 WHO Expert Review took it upon themselves to drive the agenda. Researchers and policy-makers from this group were active in the drafting of the 2001 call for research and many of these same researchers either ran or advised on the individual trials. These same policy-makers then played a central role in passing the Global Strategy for the Prevention and Control of STI at the World Health Assembly [23], thereby priming the stage for policy change with a renewed international focus on STIs. When questioned on this club-like camaraderie among key contributors to the Montreux meeting, one researcher answered, “well, many of us were here [at the WHO] for the [development of the] first [syndromic management] guidelines.”

Although in many respects, at the international level, researchers and policy-makers worked in tandem, the existence of a divide between the so called ‘two-worlds’ was plainly evident through the course of the Montreux meeting. There, program managers also played a role in the decision-making process. All three groups wanted something different out of the research findings: researchers were interested in the statistical significance of the findings and implications for further studies; policy-makers wanted a recommendation for policy-change; and program managers wanted to be able to supply the communities they served with acyclovir based on the observed disease burden. One program manager voiced his/her concern directly to the researchers: “Are you going to make any recommendations? All I hear is the need for *more* research.” On this point, policy-makers agreed. All non-researchers interviewed were frustrated with the researchers for not getting to the bottom of “the so-what factor” as one interviewee termed it.

### Mechanisms of getting research into policy in Ghana

According to WHO interviewees, it often takes as long as two years, or sometimes even longer, for changes in treatment guidelines to be disseminated through national governments to practitioners working in low income countries. One WHO policy maker predicted: “we will be lucky if this [change in the syndromic management treatment guidelines] even gets to the [WHO] country offices in two years time.” For this reason, Ghanaian interviewees were asked to speculate on the likely process for policy change in regards to the HSV-2 treatment based on their extensive experience with previous changes in WHO STI treatment guidelines. Donors’ stringent funding eligibility requirements were repeatedly cited as the single strongest impetus for policy change. One program manager interviewed stated, “where there is money, we manage to get something done, otherwise we have competing priorities.” This is not surprising as Ghana’s health sector, particularly in the area of prevention, is heavily dependent on foreign aid. Therefore, donors’ interests may often dominate research agendas, programming decisions, and policy, simply due to their financial power. Another interviewee, a researcher, commented, “I am not sure to what extent we or any developing country owns the research agenda … people come from outside and say, ‘we want to look at this and we have this money.’ There is a lot of frustration and a lot of brain drain.” This domination by donor interests can therefore work in two ways: (1) if a donor pays attention to a particular issue it gains national importance, and (2) if a donor does not emphasize an issue, it may be ignored nationally to accommodate a competing priority. Program managers and even some policy-makers felt that they were unable to act upon research findings, even in cases where it was clear that change would be beneficial because “we don’t have time and we must be responsive to donors first or we cannot do anything at all,” said one policy maker. According to all interviewees, policy-makers [in Ghana] often give more weight to international research results over national research due to the wider dissemination of research findings and the potential influence on donor funding. The policy-makers went further to contend that, in the case of HSV-2 treatment, without clear evidence that acyclovir disrupts the cofactor effect between HSV-2 and HIV, they did not anticipate including acyclovir in the syndromic management cocktail, unless there was funding attached both for procurement and for drug delivery. Because the trial results did not indicate that HSV therapy was an effective HIV prevention strategy, the same interviewees highly doubted that donor organizations, many of which they feel were myopically HIV-focused, would make herpes treatment a priority.

The researchers all felt that there was great difficulty in attracting the policy-makers’ attention to this issue: “there is so much going on, it sometimes doesn’t matter how loud your voice is.” The majority of researchers interviewed went further to predict that this is a case in which formal policy change will lag behind practice and that acyclovir might be used because of the high prevalence of HSV-2 among GUD cases, provided the drugs can be made available. Practice was also a precursor to policy reform for the initial adoption of STI syndromic management itself [51], indicating that in the interest of expediency of delivery, it may be the case that practice may often precede national STI policy reform [49]. Some argued that in the case of HSV-2, in private settings and on a small scale, practice was already preceding policy, as acyclovir is available and prescribed. As noted earlier, acyclovir was not on the national essential drugs list and so not available in the public sector.

This is not to say that Ghanaian policy reform is only responsive to donors’ directives. Another mechanism through which change may occur is if an influential enough individual (a ‘policy entrepreneur’) who is either placed directly within the Ministry of Health or National HIV Control Program, and, according to one Ghanaian researcher, “who is well liked either within [the Ministry of Health] or outside [in the community],” makes a strong case for the change. This mechanism is particularly effective for getting operational research findings into policy. In order for a policy entrepreneur to bring about change, however, all interviewees agreed that conditions apply. The policy change must be seen to: (a) save money in the long run, (b) be highly visible and good for public relations, (c) be beneficial to the population at no extra cost, or (d) have any extra costs covered by donor agencies, and/or (e) have the potential to attract additional donor funding. These conditions essentially outline the prerequisites for generating political will at the national level.

Ghana’s experience, unsurprisingly, pointed to the many ways that key networks of actors – and links between them– were essential to understanding the research to policy process. Researchers interviewed mentioned their friends in policy positions as being instrumental in the researchers’ attempts to impact policy: “If I see something needs to be done and it’s not, I call my friend,” said one researcher. This dynamic constitutes a policy network, one that links researchers with well placed individuals able to influence policy. Based on the interviews, it appears that within these networks it is often a single champion of a cause (or policy entrepreneur) who carries the issue and generates internal pressure, although this may not always be the case. Many policy-makers and program managers confirmed the existence of these communities. They spoke of personal ties through either old friends or colleagues to certain research groups and cited these ties as their main taproot to the latest research. One policy-maker in Ghana recommended, “We need to make these networks more solid [as a mechanism for bringing research and policy closer together].”

## Discussion

According to the Montreux interviewees, the comparatively high profile of HIV generated the political will necessary for HSV-2 to be brought on to the international research agenda. This indicates that even within the context of the WHO, which is comparatively immune to the multiple competing priorities seen to impede national policy reform, policy change still has political elements. In order to address issues which receive little political attention, linking to a larger, more popular issue may be necessary in order to gain political traction. In addition, there was a resounding call from policy-makers and program managers for researchers to engage in helping to bridge the divide between research and practice through indicating the programmatic and policy relevance of both their research and their findings.

On the Ghanaian national policy front, multiple competing priorities appeared to influence the uptake of research findings into national policy reform. Three mechanisms emerged. The first is to bypass policy reform. Many of the researchers in Ghana also have a clinical practice. Therefore, the distance between research and practice can be shortened, as compared to the distance between research and policy. Bypassing policy as a rule will not, however, support provision of care for all who need it. The second mechanism is orientated towards policy reform and focuses on the central role of actors in the national context. Both policy networks and policy entrepreneurs (often working within this group), were described as critical to bridging the gap between research and policy. In the case of Ghana, policy networks are cited as the only functional communication channel between researchers and policy-makers. A final mechanism for influencing policy involves donors. At the national level, donor attention to a particular issue, or lack thereof, heavily influences the likelihood of policy reform. In the case of HSV-2, it was predicted that a lack of donor attention combined with competing donor priorities, would impede the inclusion of acyclovir in national syndromic management guidelines.

**Figure 1:**
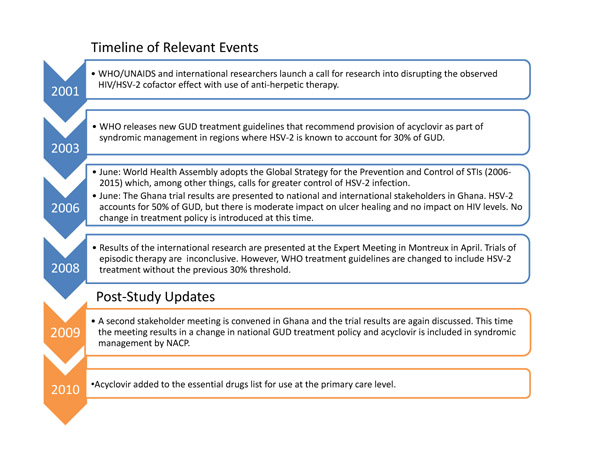
**Timeline of Relevant Events**.

Since completion of our study, further policy development has occurred in Ghana. The prediction that lack of donor interest would impede policy change proved incorrect. This appears to be because of the influence of a key policy network (linking both policy-makers and researchers) who, in 2009, met at the National AIDS Control Program to discuss implications from the findings of the Ghanaian HIV/ HSV-2 trial. During the course of the meeting, which included Ghanaian Government health officials, it was decided that acyclovir should and would be added to national GUD treatment policy. It is not clear how this meeting was convened or who drove the agenda, however, what is evident is that there was a network of interested and connected researchers and policy-makers at the heart of the process (Figure 1).

At the international level, there appears to be more formalized networks of actors influential in WHO decisions. The majority of individuals invited to attend the Expert Meeting in Montreux are actually frequent advisors to the WHO. In the case of STIs, this invitation does not appear to extend well beyond the bounds of the personalities present at the meeting, as evidenced by the observation that the same individuals had been involved in the various iterations of the STI syndromic management guidelines development.

## Conclusion

It becomes evident that in the case of HSV-2 and the changes made to the GUD syndromic management guidelines, HIV and the observed cofactor effect between HIV and HSV-2 played a pivotal role in generating the political will necessary for the creation of a policy window. Policy networks and policy entrepreneurs emerged as playing recurrent important roles. It appears that these communities and the actors that comprise them may provide the primary impetus for policy change, within both case study contexts. Evidence alone emerged as unable to influence policy without the engagement and alignment of multiple factors.

Based upon these research findings, there are several follow-on recommendations for increasing the likelihood of research uptake into policy/guidelines at both the national and international levels in the field of STIs and HIV. Within the WHO, it appears important to link with existing priorities (if possible) in order to attract attention to an otherwise marginalized issue. The entire sexual health/STI research to policy continuum lives within the apparent confines of the WHO STI policy network. This is a formalized community and one that is recognized for its contribution to policy change. Researchers may wish to proactively target this network, particularly in communicating the practical application of their findings (the “so what factor” of their work). Ideally, such communication would begin at the outset of research work and continue throughout the duration of any study, thereby building up to any potential recommendations for change, and establishing legitimacy of the researchers to the network members when their findings become available.

In the case of Ghana, the policy network is entirely informal, and yet is still a powerful contender in affecting policy change. The existence of this group presents an opportunity to disseminate study findings and exert pressure on policy-makers in an effective manner. In this way, often difficult to reach policy-makers may be included in research from the outset of a study, instead of as an endpoint for the presentation of research findings. In this way, policy-makers may also better understand the policy implications of research findings particularly in the case of research conducted locally. Also key to the national research-to-policy continuum are donors. Donor engagement around policy change is a difficult issue. Donor power emerged as a consistent theme in the Ghanaian case study, and there is a high likelihood that this finding can be extrapolated to other health areas and potentially other low-income countries. The possibilities then for ethical donor engagement in research-uptake at the national level, and particularly in the case of research conducted locally, deserves further study.

## Competing interests

The authors declare that they have no competing interests.

## Role of authors

H Burris and P Mayaud conceived and designed the study with contributions from Y Adu-Sarkodie and J Parkhurst. H Burris implemented the field survey with support from Y Adu-Sarkodie. Analysis and interpretation of data was first conducted by H Burris and reviewed by J Parkhurst and P Mayaud. The initial draft of the manuscript was written by H Burris and revised by all authors who approved its final version.

## Ethical approval

The study was approved by the Research Ethics Committee of the London School of Hygiene & Tropical Medicine and the Ethics Board of the School of Medical Sciences, Kwame Nkrumah University of Sciences & Technology, Kumasi, Ghana. Consent was obtained orally under conditions of anonymity in Switzerland and formal written consent was obtained from all interviewees in Ghana.

## Presentation

This work was presented as a poster presentation at the Bi-Annual Conference of the International Society for Sexually Transmitted Diseases Research (ISSTDR), London, 28 June -1 July 2009 (Abs P4.158).
